# The mediating role of resilience in the effects of physical exercise on college students’ negative emotions during the COVID-19 epidemic

**DOI:** 10.1038/s41598-021-04336-y

**Published:** 2021-12-31

**Authors:** Xuening Li, Huasen Yu, Ning Yang

**Affiliations:** 1 CNRS, Brain and Cognition Research Center (CerCo), Université de Toulouse 3, Toulouse, France; 2 College of Physical Education and Health, East China Normal University, Shanghai, China; 3 Institute of Physical Education, Shandong Youth University of Political Science, Jinan, China

**Keywords:** Psychology, Public health

## Abstract

Due to its suddenness and unpredictability, COVID-19 caused strife and effects on public mental health, resulting in a surge of negative emotions. The study explores the relationship between physical exercise and negative emotions in home-based college students during the COVID-19 epidemic, as well as the mediating role of resilience, thus providing a new basis for understanding the role of physical exercise in improving negative emotions in college students; A total of 1214 college students were investigated with the Physical Exercise Questionnaire, Negative Emotion Scale and Resilience Scale; Both physical exercise and resilience were significantly negatively correlated with negative emotions in college students (r = − 0.25, − 0.33, P < 0.001), and there was a significant positive correlation between physical exercise and resilience (r = 0.47, P < 0.001). Physical exercise had a direct effect on the negative emotions of college students (β = − 0.14, P < 0.001). Resilience had a partial mediating effect between physical exercise and the negative emotions of the college students, with a mediating effect value of 0.14 and a mediating effect contribution rate of 50.00%; The study found that physical exercise not only directly affected the negative emotions of college students but also improved their resilience by slowing down their negative emotions and promoting their mental health.

## Introduction

The novel coronavirus disease 2019 (COVID-19), a hideous pandemic that emerged in 2019, has caused governments of heavily affected countries to limit activities outside the home to essential tasks or to impose stay-at-home orders on their citizens. As of 7 September 2021, 221,134,742 confirmed cases, including 4,574,089 deaths, have been reported to the World Health Organization (WHO)^1^. Due to its suddenness, contagion, unpredictability, and the associated information overload, COVID-19 has caused tremendous strife for public mental health, which has resulted in a surge of negative emotions including anxiety, depression, and lower mental well-being^2,3^. For students, a combination of extended vacations, long stays at home, reduced social activities, and changes in the environment have had a significant impact on their studies and personal lives, exacerbating the occurrence of negative emotions; these consequences have likely increased anxiety and depressive symptoms such as helplessness, hopelessness and worry^4–6^. An online survey administered to 405 Chinese college students revealed that the prevalence of anxiety and depressive symptoms was 44.0% and 42.2%, respectively^7^. A cross-sectional web-based survey suggested that college students living in Bangladesh have experienced heightened depression and anxiety, as well as an unparalleled growth of depression and anxiety under the current global pandemic situation compared to earlier research. In fact, approximately 15% of college students living in Bangladesh had moderately severe depression, whereas 18.1% suffered from severe anxiety^8^. Varma et al. conducted a global cross-sectional survey, and found that 59% of respondents met the criteria for clinically significant anxiety, and 39% reported moderate depressive symptoms^9^. Individuals’ mental health in many countries has deteriorated compared to pre-COVID-19 trends^10–15^. According to emerging international evidence, anxiety and depression were common during the early stages of the COVID-19 epidemic^16,17^. As such, it is necessary to explore scientific strategies to prevent anxiety and depression in college students who have been confined at home during the COVID-19 epidemic.

In the present study, physical exercise refers to leisurely physical activity that could improve cardiorespiratory capacity, muscle strength, body composition, and flexibility^18^. There is growing evidence that individual emotions are susceptible to physical exercise^19,20^. In particular, physical exercise has great potential in preventing and treating depression and anxiety^21–23^. Regular physical exercise can significantly reduce negative emotions such as pessimism, tension, anxiety, and restlessness^24,25^. Knöchel et al. proposed that physical exercise could relieve symptoms of depression and would be an innovative approach to improving quality of life and reducing physical illness^26^. Kim and Leem suggested that chronic exercise may improve the disturbance of hippocampal 5HT1A-regulated cAMP/PKA/CREB signalling in the depressed brain, thereby exerting an antidepressant effect^27^. In line with previous studies, Zschucke et al. indicated that physical exercise can activate the hippocampus, inactivate the prefrontal cortex, and inhibit the cortisol response to the Montreal Imaging Stress Task (MIST)^28^. Recent studies have largely described the positive role exerted by physical exercise to counteract prevalent anxiety and depression in self-isolated people during the COVID-19 epidemic^29–31^. They reported that leisure-time physical activity is more closely associated with positive mental health. Furthermore, 15–30 min a day of moderate to vigorous physical activity implies lower odds of prevalent depressive and anxiety symptoms. Importantly, prolonged stays at home can reduce physical exercise levels, which leads to a significant, negative impact on mental health and well-being^32,33^. Based on these scientific evidences, regular physical exercise is a key strategy for relieving anxiety and depression in college students, especially during the COVID-19 epidemic.

Resilience can be defined as the potential or ability to adapt effectively in the face of setbacks, which is crucial for mental and physical health^34,35^. Anxiety and depression among college students increased substantially during the epidemic^36,37^. Nevertheless, resilient individuals are equipped with the ability to handle negative emotions and crises successfully^38–40^ and experience less psychological pain, thus exhibiting better mental health^41^. Resilience is negatively related to anxiety and depression^42–44^. Additionally, resilience may be an important factor in reducing depressive symptoms, internalising problems, externalising problems, and lowering general psychological distress, which helps individuals to maintain healthy and stable psychological states^45,46^. There is scientific consensus that physical exercise can improve one’s level of resilience. Moreover, physical exercise is a critical path to promote resilience^41,47^. Accumulated evidence has found that brain-derived neurotrophic factor levels increase significantly with physical exercise, which protects neurons in the striatum of the brain and hippocampus under stress, thus enhancing resilience^48^. People with high levels of physical exercise are more likely to develop resilience^49^. Further, individuals who maintained regular physical exercise during confinement reported significantly high levels of resilience^50,51^. Consequently, physical exercise could alleviate anxiety and depression by improving the level of resilience among college students during the COVID-19 epidemic. Based on these evidences, resilience could be an internal mechanism that plays a mediating role in the relationship between physical exercise and negative emotions.

Although previous studies have shown that physical exercise, negative emotions and resilience are positively correlated with each other, they have not been examined as an interactive system. Therefore, we aimed to investigate the internal psychological mechanism of the effect of physical exercise on negative emotions in college students during the COVID-19 epidemic from the perspectives of cognition and coping methods. This research not only enriches the literature on sports psychology, but also provides new ways of thinking about prevention and intervention relative to anxiety and depression among college students.

## Methods

### Ethical statement

This study was approved by the Ethical Committee of the University of Shandong Youth University of Political Science. All participants were given a brief introduction to the study and informed of its purpose, as well as declarations of anonymity and confidentiality before participating, and provided informed consent. We conducted this study in accordance with the latest revised ethical guidelines of the Declaration of Helsinki.

#### Study design and participants

The participants completed an online questionnaire survey while isolating at home during the COVID-19 epidemic. The questionnaire survey was sent via a professional platform called ‘Wenjuanxing’. Moreover, the form link was shared by the research group members and different student groups on We Chat. We collected data were collected from 15 to 20 March 2020. All universities were closed and all college students stayed at home during the epidemic. Thus, we used a simplified cluster sampling method based on a random selection of 1,260 students from three universities in the provinces of Shandong, Liaoning and Jilin. The sample comprised 210 students majoring in liberal arts and 210 students majoring in science from each university for each grade. Among the 1,260 college students who completed the questionnaire surveys, we excluded 46 because they provided incomplete or faulty data, yielding a final sample of 1,214 students. The return rate was 97.12%. The resulting 1,214 participants completed the entire questionnaire survey. There was a minor imbalance in gender 506 males (41.68%) and 708 females (58.32%)—and the mean participant age was 19.99 (SD=1.73).

#### Physical Exercise Questionnaire

To assess the effect of physical exercise in the Chinese population^52^, Wu et al. revised items from an existing measure developed for Chinese college students^53^. The final scale comprised 8 items with a score ranging from 0 to 40, and entailed a 5-point scale ranging from 1 (totally disagree) to 5 (totally agree); it contained 2 dimensions: exercise adherence and exercise commitment. We summed the individual item scores to produce the total scores, with higher scores indicating greater physical exercise. However, in the present study, one item (work by fits and starts) under exercise adherence had a factor loading with an absolute value of 0.29, and was rejected because it did not meet the standard that factor loadings must have absolute values of not less than 0.40^54^. Thus, we deleted the data for this item while performing statistical analysis. The Cronbach’s alpha in the present research was 0.93, ranging from 0.83 to 0.91 across the two subscales.

#### Depression-Anxiety Scale

We used the 21-item assessment, which was extracted from the original 42-item scale, as a brief symptoms inventory to assess the previous week^55,56^. The scale was revised in 2012 to accommodate the Chinese population and then pilot tested. The revised scale includes three subscales: anxiety (7 items, α= 0.82), depression (7 items, α= 0.75), and pressure (7 items, α= 0.80); it has good reliability, validity, and applicability among Chinese adults^57^. We scored each item on a 4-point scale ranging from 0 (totally disagree) to 3 (totally agree). The total scores range from 0 to 42, with higher scores showing more severe negative emotions. However, we only used two subscales (anxiety and depression), to assess individual negative emotions. The Cronbach’s alpha of this measure in the present research was 0.93, ranging from 0.86 to 0.87 across the two subscales.

#### Resilience Scale (CD-RISC)

In 2007, Yu and Zhang modified the Connor-Davidson Resilience Scale, to measure resilience^58^, which has acceptable reliability and validity among Chinese individuals. The CD-RISC is composed of 25 items with a score ranging from 0 to 100; assesses tenacity (13 items, α=0.88), strength (8 items, α=0.80), and optimism (4 items, α= 0.60)^59^. Each item is scored on a 5-point scale ranging from 0 (never) to 4 (always), with higher scores indicating greater resilience. Among the 25 items collected to explore the correlation in the present study, one item (had to act on a hunch) was rejected due to a low factor loading (absolute value 0.20). The specific reason has been described above. The Cronbach’s alpha for the CD-RISC in the present study was 0.92, and for each of the three subscales, it ranged from 0.67 to 0.88.

#### Statistical analysis

We gathered the data and processed them using Excel software. We performed descriptive statistics, correlation analysis, and t-tests with SPSS version 22.0. Further, we adopted a statistical significance level of p<0.05. All variables are shown as the mean and standard deviation (SD). We used structural equation modelling (SEM) to investigate the mediating role of resilience in the impact of physical exercise on negative emotions, assuming that resilience and physical exercise from college students would be directly and indirectly associated with negative emotions. We carried out SEM analysis using the AMOS statistical program^60^. SEM is an appropriate method for investigating the relative effects of multiple predictors on multiple outcomes and controlling for measurement errors^61^. We first conducted exploratory factor analysis (EFA) for each scale (physical exercise, resilience, negative emotions) to check their dimensionality. Factors distilled from the EFA and filtered at a minimum loading threshold of 0.40 were the latent variables for the structural equation models^62^. We verified the measurement model using confirmatory factor analysis (CFA). In addition, we used the root mean square error approximation (RMSEA) to evaluate the model fit. We considered a RMSEA value of less than 0.05 to be a ‘close fit’, and a value between 0.05 and 0.08 to be an ‘acceptable fit’; RMSEA values larger than 0.1 signalled a ‘poor fit’ ^63^. The normal fit index (NFI), comparative fit index (CFI), non-normed fit index (NNFI), and adjusted goodness-of-fit index (AGFI) were also important indices; we considered a model with values greater than 0.90 to be a good one. Therefore, we focused on RMSEA, NFI, CFI, NNFI, and AGFI.

## Results

### Correlation analysis

Table 1 presents the descriptive statistics and Pearson correlation coefficients of physical exercise, negative emotions, and resilience. As shown in Table 1, the mean physical exercise of participants was low, at 23.01 (SD = 5.61). The mean scores on exercise adherence and exercise commitment were 9.86 (SD = 2.63) and 13.15 (SD = 3.30), respectively. The resilience of college students averaged 59.16 (SD = 14.62). The mean tenacity, strength, and optimistic scores were 29.83 (SD = 8.53), 8.15 (SD = 2.40), and 21.37 (SD = 5.12), respectively. Participants reported negative emotions with a mean score of 6.83 (SD = 7.24), and the mean scores of anxiety and depression were 3.52 (SD = 3.65) and 3.31 (SD = 3.77), respectively, on a 21-point scale. The results revealed that physical exercise was negatively associated with negative emotions (r = − 0.25, P < 0.001), and positively correlated with resilience (r = 0.47, P < 0.001). Additionally, resilience negatively correlated with negative emotions (r = − 0.33, P < 0.001). There was a high degree of correlation among all variables. These results supported further testing of the mediation and moderated mediation models.

**Table 1:** Descriptive statistics and Pearson correlations coefficient of physical exercise, negative emotions, and resilience, N = 1214.

Variables	M ± SD	1	2	3
1. Physical exercise	23.01 ± 5.61	1.00	0.47**	− 0.25**
2. Resilience	59.16 ± 14.62	0.47**	1.00	− 0.33**
3. Negative emotion	6.83 ± 7.24	− 0.25**	− 0.33**	1.00

### Mediation analysis

**Figure 1:**
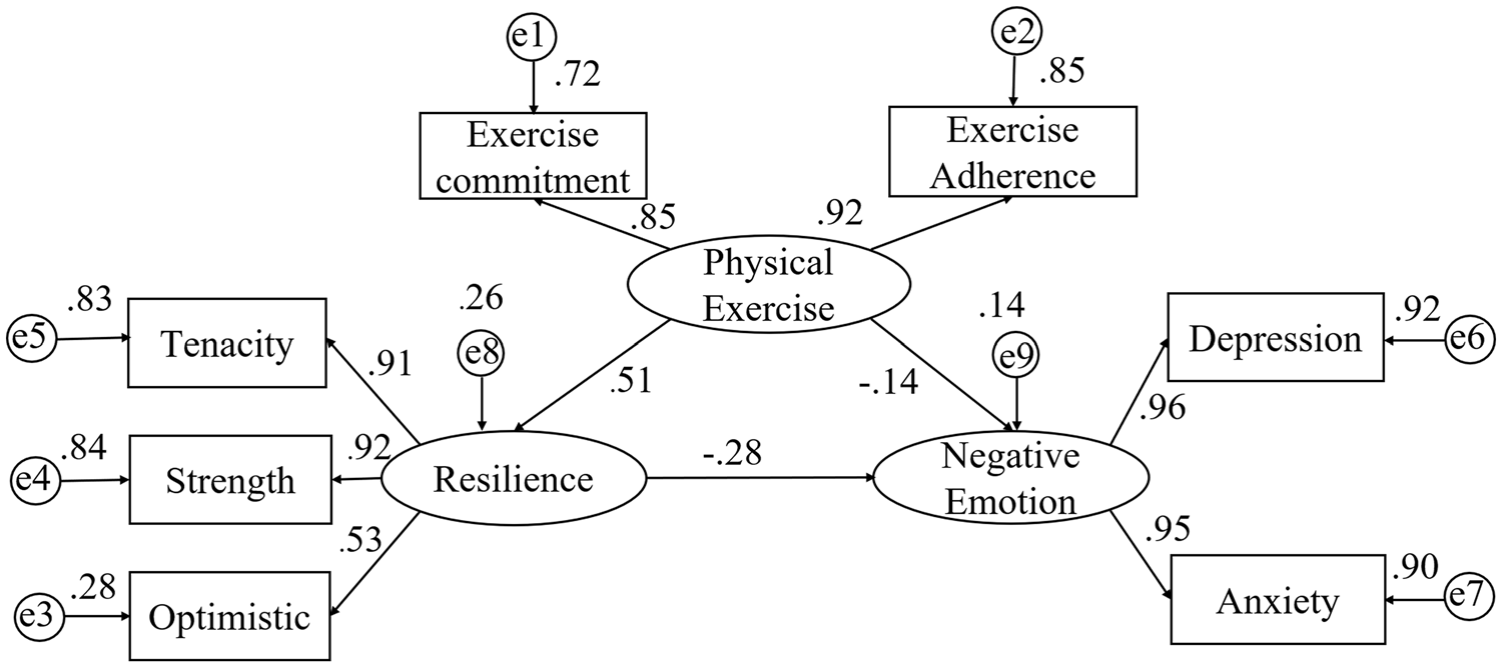
A structural equation model testing resilience factors—tenacity, strength, and optimistic—as mediators of the physical exercise and negative emotion.

The results in Table 1 showed that there was a significant correlation among physical exercise, resilience, and negative emotions, which provided a basis for testing the mediating role of resilience. Therefore, we employed the AMOS 23.0 and the maximum-likelihood method to construct the model for analyzing the relation of variables. As shown by the CFA results in Fig. 1: χ^2^/df = 4.698, GFI = 0.988, CFI = 0.993, AGFI = 0.970, NFI = 0.991, IFI = 0.993, RMSEA = 0.055, which indicated that the measurement model provided an acceptable fit for the data.

### Mediation effect

The results presented in Fig. 1 indicated a significant indirect effect of resilience in mediating the relationship between physical exercise and negative emotions. Then, we used the bootstrap method to test the mediation effect of the structure model. The results of SEM analysis, as shown in Table 2, demonstrated that physical exercise had a direct and negative effect on negative emotions (β = − 0.14, t = − 0.18, p < 0.01), and the 95% confidence interval was from − 0.32 to − 0.19. Resilience was negatively related to negative emotion (β = − 0.25, t = − 0.71, p < 0.01), and the 95% confidence interval was from − 0.33 to − 0.16. Physical exercise had a significant and positive predictive effect on resilience (β = 0.51, t = 0.23, p < 0.01). Additionally, bootstrap confidence intervals were used to estimate the mediation effect. The results showed that the mediation effect is 0.14, the 95% confidence interval is from − 0.19 to − 0.10, and the confidence interval did not include 0. The total effect of physical exercise on negative emotions was 0.28. Therefore, resilience played a partial mediating role connecting physical exercise and negative emotions. In addition, the mediation effect accounted for 50.00% of the total effect.

**Table 2:** Resilience moderates the effect of physical exercise on negative emotions.

Variables	Effect	Estimate	Bias-corrected 95% CI	
			Upper limit	Lower limit
Physical exercise → negative emotion	Total effect	− 0.28	− 0.34	− 0.21
Physical exercise → negative emotion	Direct effect	− 0.14	− 0.21	− 0.06
Physical exercise → negative emotion	Indirect effect	− 0.14	− 0.21	− 0.10
Physical exercise → resilience	Direct effect	0.51	0.44	0.56
Resilience → negative emotion	Direct effect	− 0.29	− 0.36	− 0.19

## Discussion

The correlation analysis indicated that physical exercise was negatively correlated with negative emotions among home-based college students during a public health emergency; that is, higher levels of physical exercise were associated with fewer negative emotions, which is consistent with findings from previous studies^64–66^. The structural equation model suggests that physical exercise is a negative predictor of negative emotions and has a significant direct effect, meaning that the occurrence of anxiety and depressive symptoms may have been influenced by low physical activity in home-based college students during the COVID-19 epidemic. The results provide additional support for the well-documented, cross-sectional link between physical exercise and negative emotions^5,67^. According to the hypothesis on the psychological impact of physical exercise, the most direct effect outcome of exercise is that it gives individuals a sense of pleasure and joy. The increase in positive emotions reduces the burden of negative ones^68^. Frequent exercise and participation in sports contribute to greater well-being and lower levels of anxiety and depressive symptoms in both sexes^69^. Physical exercise has significant anti-anxiety and anti-depressive effects, and is conducive to improving mental health^70,71^. Besides, moderate physical activity could amortise its negative effects on psychological health and lead to a more positive mental state^72^. In addition, physical exercise is a positive, effective means of health promotion, and an increase in exercise can improve the brain’s emotional processing ability to relieve anxiety and depression^73^.Thus, based on the abovementioned mechanisms, physical exercise could be an intervention to treat anxiety and depression by improving a variety of physiological and psychological factors.

Our results show that resilience played a partially intermediary role in connecting physical exercise to negative emotions in college students, which is consistent with the findings of previous studies^74^. On the one hand, the structural equation outcomes revealed that physical exercise had a significant, positive predictive effect on college students’ resilience, which supported the findings of Lines et al.^75^ In terms of the relationship between physical exercise and resilience some researchers believe that physical exercise is a protective factor^76^. The internal factors that influence resilience, including cognitive, problem-solving, interpersonal and emotional skills, as well as increasing individuals’ internal resources can generate greater resilience^77^. In particular, when one encounters troublesome circumstances or challenges, there will be an interaction between the individual and the environment. At this time, internal factors play a positive role in helping individuals to cope with difficulties in a positive manner. Resilience is not fixed and can potentially be strengthened by implementing certain interventions^78,79^. An experimental study of 1,546 first-year students found that physical exercise can effectively improve college students’ resilience^80^. This may be because regular participation in physical exercise can successfully reduce physiological stress levels, harmonise emotions, and improve one’s sense of self-control and state of mind, enhancing the level of resilience^81,82^. Additionally, physical exercise helps to cultivate students’ positive psychological qualities such as self-confidence, extroversion, optimism, and emotional stability. Students can obtain a sense of pleasure and satisfaction—both spiritually and mentally—through physical exercise, leading to positive changes in psychological functions. Thus, physical exercise can be used to improve the level of resilience among home-based college students.

On the other hand, the structural equation results imply that being resilient is negatively linked with experiencing anxiety and depressive symptoms. This outcome is line with previous studies, illustrating that resilience has a buffering effect on the negative emotional consequences of adverse events^83,84^. According to the dynamic model of mental resilience, resilience is a skill that can be acquired; moreover, internal resources such as control, formed during the adolescents’ developmental process, are crucial for the protective role of healthy growth^78^. As a vital component of mental health, resilience can help individuals to cope with negative emotions through optimistic attitudes that positively predict mental health^85^. The resilience model, proposed by Dray et al., indicates that people with high resilience are better able to face challenges, deal with them in more positive ways, and seek for support, and effectively solve problems^45^. Thus, individuals can better cope with negative emotions and overcome difficulties by using resilience. This positive psychological response mode is conducive to improving college students’ sense of self-control and reducing levels of depression and anxiety^86,87^. Due to the protective effect of resilience, individuals can make full use of internal resources to adapt or recover, despite adverse conditions, and to eliminate negative emotions^80^. In one study, a resilience intervention strengthened the impact of physical exercise on anxiety and depression^88^. Based on these evidences, it is clear that resilience significantly moderates the relationship between physical exercise and depression, and anxiety. As shown by the above results, there is a strong correlation between physical exercise and negative emotions. These findings provide the best support for claims that routine physical exercise has protected against negative emotions during the COVID-19 epidemic. Hence, physical activity should be encouraged to reduce post-traumatic anxiety and depressive symptoms. This intervention is fairly easy to obtain online without requiring in-person contact, which is especially vital during the COVID-19 epidemic.

However, we faced several limitations. First, our study was a cross-sectional one. Although this allowed us to objectively reflect the relationship between different variables, we were unable to establish an exact causal relationship. Second, the sample lived in three provinces (Jilin Province, Liaoning Province, and Shandong Province) and was not collected as a representative sample. The participants only had bachelor’s degree, which is not representative of college students in China. Third, the physical exercise questionnaire was not recognised, but modified by Chinese scholars. More specifically, it is only applicable to Chinese students. The use of larger, validated measurement scales could better determine how specific exercise types such as (exercise frequency, intensity, and time) affect anxiety and depression. Finally, there may still be some potential moderators that we have not considered.

## Conclusions

We found physical exercise to be associated with less anxiety and depression during the COVID-19 epidemic. Additionally, resilience plays a mediating role in the relationship between physical exercise and negative emotions. Thus, physical exercise focused on building resilience might be a cost-effective strategy to reduce anxiety and depression during the COVID-19 epidemic Future studies should combine horizontal and vertical interventions to identify other potentially mediating variables to explore internal psychological mechanisms.
